# Betel Nut Chewing Was Associated with Obstructive Lung Disease in a Large Taiwanese Population Study

**DOI:** 10.3390/jpm11100973

**Published:** 2021-09-28

**Authors:** Chao-Hsin Huang, Jiun-Hung Geng, Da-Wei Wu, Szu-Chia Chen, Chih-Hsing Hung, Chao-Hung Kuo

**Affiliations:** 1 Department of Post Baccalaureate Medicine, Kaohsiung Medical University, Kaohsiung 807, Taiwan; dragonc09931227@gmail.com; 2 Department of Urology, Kaohsiung Municipal Siaogang Hospital, Kaohsiung Medical University, Kaohsiung 812, Taiwan; u9001090@gmail.com; 3 Department of Urology, Kaohsiung Medical University Hospital, Kaohsiung Medical University, Kaohsiung 807, Taiwan; 4 Department of Internal Medicine, Kaohsiung Municipal Siaogang Hospital, Kaohsiung Medical University, 482, Shan-Ming Road, Hsiao-Kang District, Kaohsiung 812, Taiwan; u8900030@yahoo.com.tw (D.-W.W.); kjh88kmu@gmail.com (C.-H.K.); 5 Division of Pulmonary and Critical Care Medicine, Department of Internal Medicine, Kaohsiung Medical University Hospital, Kaohsiung Medical University, Kaohsiung 807, Taiwan; 6 Division of Nephrology, Department of Internal Medicine, Kaohsiung Medical University Hospital, Kaohsiung Medical University, Kaohsiung 807, Taiwan; 7 Faculty of Medicine, College of Medicine, Kaohsiung Medical University, Kaohsiung 807, Taiwan; 8 Research Center for Environmental Medicine, Kaohsiung Medical University, Kaohsiung 807, Taiwan; pedhung@gmail.com; 9 Department of Pediatrics, Kaohsiung Medical University Hospital, Kaohsiung Medical University, Kaohsiung 807, Taiwan; 10 Department of Pediatrics, Kaohsiung Municipal Siaogang Hospital, Kaohsiung Medical University, Kaohsiung 812, Taiwan; 11 Division of Gastroenterology, Department of Internal Medicine, Kaohsiung Medical University Hospital, Kaohsiung Medical University, Kaohsiung 807, Taiwan

**Keywords:** obstructive lung disease, betel nut chewing, cumulative dose, Taiwan biobank

## Abstract

The prevalence of betel nut chewing in Taiwan is high at approximately 7%, however, few studies have evaluated the relationship between betel nut chewing and lung disease. Therefore, the aim of this study was to investigate associations between betel nut chewing and lung function in 80,877 participants in the Taiwan Biobank (TWB). We further investigated correlations between betel nut chewing characteristics such as years of use, frequency, daily amount, and accumulative dose, with obstructive lung disease. We used data from the TWB. Lung function was assessed using spirometry measurements of forced vital capacity (FVC) and forced expiratory volume in 1 s (FEV1). The participants were classified into normal lung function and obstructive lung function (FEV1/FVC < 70%) groups. The participants were asked questions about betel nut chewing, including years of use, frequency, and daily amount. After multivariable analysis, betel nut chewing (odds ratio [OR] = 1.159; *p* < 0.001) was significantly associated with FEV1/FVC < 70% in all participants (*n* = 80,877). Further, in the participants who chewed betel nut (*n* = 5135), a long duration of betel nut chewing (per 1 year; OR = 1.008; *p* = 0.012), betel nut use every day (vs. 1–3 days/month; OR = 1.793; *p* = 0.036), 10–20 quids a day (vs. <10 quids; OR = 1.404; *p* = 0.019), 21–30 quids a day (vs. <10 quids; OR = 1.662; *p* = 0.010), ≥31 quids a day (vs. <10 quids; OR = 1.717; *p* = 0.003), and high cumulative dose (per 1 year × frequency × daily score; OR = 1.001; *p* = 0.002) were significantly associated with FEV1/FVC < 70%. In this large population-based cohort study, chewing betel nut was associated with obstructive lung disease. Furthermore, a long duration of betel nut chewing, more frequent use, higher daily amount, and high cumulative dose were associated with obstructive lung disease. This suggests that preventing betel nut chewing should be considered to reduce obstructive lung disease in Taiwan.

## 1. Introduction

Chronic lung disease has attracted increasing attention due to the high global prevalence of around 545 million people, among which chronic pulmonary obstructive disease (COPD) has the highest global prevalence of 3.9% [1]. This high burden may be related to environmental exposure and inhalation of toxins. Long-term exposure to noxious particles causes irreversible chronic inflammation resulting in small airway fibrosis, mucus hypersecretion, tissue destruction, increased lung compliance, and progressive airflow obstruction [2]. These pathological changes impair perfusion and lead to the development of COPD. Acute exacerbations of COPD worsen impaired perfusion and significantly contribute to comorbid conditions such as skeletal muscle wasting, cachexia, lung cancer, and mortality [3].

Betel nut chewing is one of the leading causes of oral cancer in Asia. Approximately 600 million people chew betel nut worldwide, mostly in South Asia, East Africa, and Western Pacific regions [4]. People chew and become addicted to betel nut due to a sense of satisfaction and restoration of alertness, which are achieved through the inhibition of gamma-aminobutyric acid uptake and upregulation of catecholamines [5]. Although betel nut chewing induces euphoria, it also has detrimental effects on health, such as leukoplakia, submucous fibrosis, oral cancer, oral microbiome imbalance, and pharyngeal cancer [6]. In 2004, betel nut was recognized by the International Agency for Research on Cancer as a group 1 human carcinogen [7]. In addition, betel nut chewing has been associated with metabolic syndrome, cardiovascular abnormities such as arterial stiffness and coronary artery disease, and the growth of colorectal polyps [8,9,10]. Although many studies have investigated the relationships between betel nut and extrapulmonary abnormalities, little is known about the effects of betel nuts on lung function. Wang et al. reported that betel nut chewing caused the release of eotaxin-1 induced by arecoline, a major alkaloid in betel nuts, which can cause asthma [11]. In addition, Lin et al. suggested that betel nut chewing and smoking were both associated with obstructive lung disease [10]. Possible pathological mechanisms of betel nut chewing include dysregulation of proinflammatory cytokines and excessive deposition in connective tissue [12]. However, the association between betel nut chewing and obstructive lung disease remains poorly defined.

Therefore, the aim of this study was to investigate associations between betel nut chewing and lung function in 80,877 participants in the Taiwan Biobank (TWB). We further investigated correlations between the characteristics of betel nut chewing such as years of use, frequency, daily amount, and accumulative dose with obstructive lung function.

## 2. Methods

### 2.1. Ethics Statement

The TWB was granted ethical approval by the Institutional Review Board on Biomedical Science Research, Academia Sinica, Taiwan, and the Ethics and Governance Council of the TWB. According to institutional requirements, all of the registered subjects gave written informed consent. Moreover, this study was conducted according to the Declaration of Helsinki under the approval of the Institutional Review Board of Kaohsiung Medical University Hospital (KMUHIRB-E(I)-20180242).

### 2.2. The TWB

The TWB is the largest government-backed biobank in Taiwan, and it contains lifestyle and genomic data of community-based volunteers aged between 30 and 70 years with no prior history of cancer [13,14]. Ethical approval for the TWB was given by the Institutional Review Board on Biomedical Science Research, Academia Sinica, Taiwan, and the Ethics and Governance Council of the TWB. All of the registered subjects are asked to complete in-person questionnaires, undergo a physical examination, and provide a blood sample after providing written informed consent. Subsequently, their data including lifestyle factors, personal factors, body height, and body weight are recorded in the TWB.

We defined regular exercise as doing at least 30 min of physical activities including cycling, yoga, water, or other types of sports, running, hiking, and indoor exercise three times a week. Manual labor related to work was not classified as exercise in this study.

### 2.3. Study Data

For each participant, we recorded data on age, sex, smoking/exercise habits, and whether they had a history of diabetes mellitus, hypertension, asthma, and emphysema or bronchitis, which were self-reported. Body mass index (BMI) was calculated as body weight/body height (kg/m^2^). Systolic blood pressure and diastolic blood pressure measurements were also recorded. BMI, systolic and diastolic blood pressures were measured by trained researchers. In addition, the following laboratory data were recorded at baseline: total cholesterol, low-density lipoprotein cholesterol (LDL-C), high-density lipoprotein cholesterol (HDL-C), white blood cell count, hemoglobin, fasting glucose, triglycerides, uric acid, and estimated glomerular filtration rate (eGFR). eGFR was calculated using the Modification of Diet in Renal Disease 4-variable equation [15].

### 2.4. Assessment of Betel Nut Chewing

The participants were asked ‘Have you ever chewed betel nut?’ If they answered ‘Yes’, then they were asked to answer the following questions:

1. ‘How many years have you chewed betel nut?’
2. ‘How often do you chew betel nut?’ (frequency)
  1–3 days/month (score = 1);
  1, 2 days/week (score = 2);
  3–5 days/week (score = 3);
  Every day (score = 4).

1. ‘How many quid a day?’ (daily amount)
  <10 quids (score = 1);
  10–20 quids (score = 2);
  21–30 quids (score = 3);
  ≥31 quids (score = 4).
2. Cumulative dose = years of chewing betel nut*frequency score*daily score.

### 2.5. Spirometry Measurements

**Figure 1:**
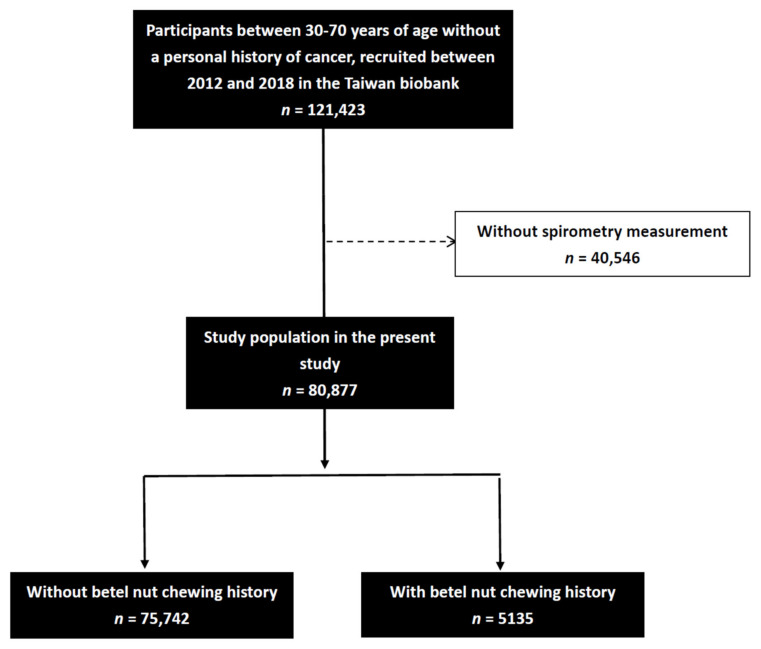
Flowchart of study population.

Data on forced vital capacity (FVC) and forced expiratory volume in 1 s (FEV1) and were recorded. The technical standards of the American Thoracic Society/European Respiratory Society were followed for all spirometry tests, which were performed by a trained technician with a MicroLab spirometer and Spida 5 software (Micro Medical Ltd., Rochester, Kent, UK) [16]. Each participant underwent lung function tests three times (each had differences within 5% or 100 mL and met quality standards). The best result of these three tests was used in further analyses. FEV1/FEV1% predicted and FVC/FVC% predicted were calculated as their values divided by reference values based on the general population according to age, sex, height, and Asian ethnicity. Spirometry software was used to calculate the percent-predicted values. FEV1/FVC < 70% was defined as obstructive lung function. Of the 121,423 screened participants, 40,546 did not have complete spirometry data and were excluded from the study. The remaining 80,877 participants who had complete spirometry measurements were then included (Figure 1).

### 2.6. Statistical Analysis

SPSS version 26.0 for Windows (SPSS Inc. Chicago, IL, USA) was used for all statistical analyses. Data are presented as percentages or the means ± standard deviations. Between-group differences in continuous variables were examined using the independent *t*-test, and between-group differences in categorical variables were examined using the chi-square test. Associations between betel nut chewing and its characteristics with FEV1/FVC < 70% were examined using multivariate logistic regression analysis. Moreover, multivariable linear regression analyses were used to identify associations between betel nut chewing and FEV1/FVC. A *p*-value < 0.05 was considered to be statistically significant.

## 3. Results

Of the 80,788 enrolled participants (50,723 females; 30,154 males), the mean age was 49.9 ± 10.9 years. The participants were divided into normal lung function (FEV1/FVC ≥ 70%; *n* = 65,199, 80.6%) and obstructive lung function (FEV1/FVC < 70%; *n* = 15,678; 19.4%) groups.

### 3.1. Comparisons of Clinical Characteristics between the Normal Lung Function and Obstructive Lung Function Groups

A comparison of the clinical characteristics between the two groups is shown in Table 1. Compared to the participants with FEV1/FVC ≥ 70%, those with FEV1/FVC < 70% were older, predominantly female, had a lower prevalence of asthma, higher prevalence of emphysema or bronchitis, higher prevalence of regular exercise, lower BMI, higher white blood cell count, lower hemoglobin, lower triglycerides, lower total cholesterol, lower eGFR, and lower FEV1/FVC, FVC, and FEV1.

### 3.2. Correlations between Chewing Betel Nut and FEV1/FVC < 70% in All Participants

Table 2 shows the determinants of FEV1/FVC < 70% in all study participants (*n* = 80,877) in multivariable logistic regression analysis. After adjusting for betel nut chewing, smoking history, regular exercise, sex, age, diabetes mellitus, hypertension, emphysema or bronchitis, asthma, BMI, white blood cell count, hemoglobin, fasting glucose, triglycerides, total cholesterol, HDL-C, LDL-C, eGFR and uric acid, betel nut chewing (odds ratio [OR] = 1.159; 95% confidence interval [CI] = 1.072–1.254; *p* < 0.001), old age (*p* < 0.001), female sex (*p* < 0.001), no asthma history (*p* = 0.001), emphysema or bronchitis history (*p* = 0.022), regular exercise (*p* < 0.001), low BMI (*p* < 0.001), high white blood cell count (*p* < 0.001), low hemoglobin (*p* < 0.001), low triglycerides (*p* < 0.001), high total cholesterol (*p* = 0.034), low HDL-C (*p* < 0.001), high eGFR (*p* < 0.001), and high uric acid (*p* < 0.001) were significantly associated with FEV1/FVC < 70%.

We further performed multivariable linear regression analysis in all study participants to identify associations between betel nut chewing history and FEV1/FVC. Betel nut chewing history (unstandardized coefficient β, −1.465; 95% CI, −1.992 to −0.937; *p* < 0.001) was significantly associated with low FEV1/FVC.

### 3.3. Comparisons of Betel Nut Chewing Characteristics among the Participants Who Chewed Betel Nut According to FEV1/FVC ≥ 70% or <70%

Table 3 shows comparisons of betel nut chewing characteristics among the participants who chewed betel nut according to FEV1/FVC ≥ 70% or <70% (*n* = 5135). Compared to the participants with FEV1/FVC ≥ 70%, those with FEV1/FVC < 70% had chewed betel nut for longer, chewed betel nut more frequently, chewed more quids a day, and had a higher cumulative dose.

### 3.4. Correlations between Betel Nut Chewing Characteristics with FEV1/FVC < 70% in the Participants Who Chewed Betel Nut

The factors associated with FEV1/FVC < 70% in the participants who chewed betel nut (*n* = 5135) in multivariable logistic regression analysis are shown in Table 4. After multivariable adjustments for betel nut chewing characteristics, smoking history, sex, age, diabetes mellitus, emphysema, asthma, hypertension, regular exercise, BMI, white blood cell count, hemoglobin, fasting glucose, triglycerides, total cholesterol, HDL-C, LDL-C, eGFR and uric acid, a long duration of betel nut chewing (per 1 year; OR = 1.008; 95% CI = 1.002–1.014; *p* = 0.012), betel nut use every day (vs. 1–3 days/month; OR = 1.793; 95% CI = 1.038–3.097; *p* = 0.036), 10–20 quids a day (vs. <10 quids; OR = 1.404; 95% CI = 1.058–1.862; *p* = 0.019), 21–30 quids a day (vs. <10 quids; OR = 1.662; 95% CI = 1.130–2.446; *p* = 0.010), ≥31 quids a day (vs. <10 quids; OR = 1.717; 95% CI = 1.199–2.459; *p* = 0.003), and high cumulative dose (per 1 year × frequency × daily score; OR = 1.001; 95% CI = 1.001–1.002; *p* = 0.002) were significantly associated with FEV1/FVC < 70% in the participants who chewed betel nut.

We further performed multivariable linear regression analysis in the participants who chewed betel nut to identify associations between betel nut chewing years and cumulative dose with FEV1/FVC. A long duration of betel nut chewing (per 1 year; unstandardized coefficient β, −0.067; 95% CI, −0.110 to −0.024; *p* = 0.002), and high cumulative dose (per 1 year × frequency × daily score; unstandardized coefficient β, −0.011; 95% CI, −0.016 to −0.005; *p* < 0.001) were significantly associated with low FEV1/FVC.

## 4. Discussion

In this study, we investigated the associations between betel nut chewing and its characteristics with lung function among 80,877 Taiwanese participants. Overall, we found that after adjusting for confounders, betel nut chewing was associated with FEV1/FVC < 70%. Furthermore, a long duration of betel nut chewing, more frequent use, higher daily amount, and high cumulative dose were correlated with FEV1/FVC < 70% in 5135 participants who chewed betel nut.

The first important finding of this study is that betel nut chewing was associated with obstructive lung disease, defined as FEV1/FVC < 70%. Although significant, the OR of FEV1/FVC < 70% was small (OR = 1.008) when years of chewing betel nut increased by 1 year. Therefore, we further investigated the frequency, daily amount, and cumulative dose of betel nut chewing in analysis. Moreover, a high cumulative dose was also correlated with FEV1/FVC < 70%. Associations between impaired lung function such as asthma and betel nut chewing have been studied since 1991. Kiyingi et al. first reported that betel nut chewing could aggravate asthma in an analysis of 61 questionnaires [17]. Taylor et al. suggested that arecoline inhalation could lead to bronchoconstriction with a decrease in FEV1 in a double-blinded study of 12 patients, six of which had asthma [18]. In addition, Wang et al. found a positive correlation between asthma and betel nut chewing in 1800 subjects, which they suggested could be due to an increase in arecoline inducing eotaxin-1 activation. Eotaxin-1 correlates to the attraction of eosinophils and Th2 lymphocytes to inflammatory sites, which in this case, was the epithelium of the airway [11]. In addition to asthma, betel nuts also associate with COPD, another common type of lung disease caused by chronic inflammation. Lin et al. conducted a cross-sectional study of 1653 participants, and their results were the first to suggest a positive correlation between betel nut chewing and obstructive lung disease [10]. The mechanism underlying the association between betel nut chewing and COPD may also be related to arecoline, which can lead to tracheobronchial hypersecretion, tracheal smooth muscle contraction, and inflammation. Kuo et al. also demonstrated that betel nut could stimulate the production of inflammatory cytokines. leading to the proliferation of bronchial smooth muscle and causing airway remodeling [19]. Taken together, we suggest that betel nut induces both asthma and COPD through eotaxin-1 activation and chronic inflammation, respectively.

Betel nuts are made of two major components: betel nut itself and slaked lime. Besides the effects of betel nut as discussed above, slaked lime can have a detrimental effect on health as well. Many studies have shown slaked lime as a carcinogen that produced reactive oxygen species [20,21]. Sazwi et al., for instance, showed that slaked lime impaired cytoprotective and anti-oxidant activities of betel nut [22]. To further analyze different components of betel nuts, Sari et al. [23] conducted a study to investigate the characteristics of betel nuts and their relationship to oral malignancy. Their results showed that slaked lime increased pH value. Besides the carcinogenicity of betel nuts, few studies have investigated slaked slime as an inflammatory regulator. However, Sarode et al. hypothesized that betel nuts alone without an inflammation mechanism or slaked slime could not lead to oral cancer [24]. Picking up on Sarode’s idea, we assumed that the potential effects of slaked lime on lung function can alter or strengthen our results. Further investigation in the next study will be designed based on this theory.

The second important finding of this study is that a high white blood cell count was associated with FEV1/FVC < 70%. A previous study reported an increased level of neutrophils in COPD patients [25]. To investigate the interactions between neutrophils and obstructive lung disease, Sapey et al. conducted a study to assess neutrophil motility in COPD patients. Their result suggested that the speed of migration of neutrophils was increased but the accuracy of direction was decreased in the presence of inflammatory factors [26]. In addition, in a study on 38 subjects, Tregay et al. found an accumulation of neutrophils in the lungs of COPD patients compared to healthy patients. Thulborn et al. suggested in a recent study that active neutrophil elastase (NE) could be a biomarker of exacerbations of COPD since an elevation of NE was observed in patients with exacerbations of COPD [27]. In a study of 40 COPD subjects, Contoli et al. showed that manifestations of symptoms such as cough and dyspnea were positively related to the number of neutrophils in the sputum. Our results are consistent with the previous studies and our first finding in that a high white blood cell count such as elevated neutrophil levels was associated with obstructive lung disease, which is related to chronic inflammation.

In this study, we also showed that fluctuations in plasma lipids such as low triglycerides, high total cholesterol, and low HDL-C were associated with FEV1/FVC < 70%. Several studies have shown that dysregulated lipid metabolism can contribute to lung diseases such as COPD. Lipids are critical components of the metabolic conduction pathway, and they have significant impacts on the regulation of inflammatory responses [28]. Disruption of lipid metabolism such as upregulation of cholesterol has been reported in COPD patients. However, the mechanism of deranged fatty acid synthesis in COPD patients remains unknown. Gunay et al. proposed that lipid indices such as high triglycerides and low HDL-C could be used as predictors of atherosclerosis in COPD patients [29]. In addition, Xuan et al. proposed that triglycerides could be a predictor of cardiovascular morbidities in COPD patients, due to high levels of triglycerides being observed in COPD patients without lipid-lowering therapy. However, in this study, we found that a low triglyceride level was associated with FEV1/FVC < 70%, which is different from previous studies. One possible explanation is the lack of medical history to eliminate the effects of lipid-lowering therapy in our study. Another explanation is that lipid metabolism is affected by various hormones, which could be dysregulated in COPD patients, thereby causing fluctuations in lipid profiles. Some studies have suggested that hormones such as leptin or lipid metabolism pathways may be involved in patients with COPD [30]. Further studies are needed to clarify the correlations between lipid profiles and COPD.

Another interesting finding of this study is that a high level of uric acid was associated with FEV1/FVC < 70%. Kahnert et al. suggested that uric acid could be used as a biomarker in COPD patients in an analysis of the German COPD cohort COSYCONET due to a positive correlation between a higher uric acid level and impaired lung function such as an increased severity of exacerbation and decreased FEV1 level. [31]. A meta-analysis by Wattanachayakul et al. also found an average 1 mg/dl higher uric acid level in COPD patients than in healthy individuals, whereas Peioto et al. observed a decrease in uric acid level in patients with a low purine diet [32]. These results suggest that hyperuricemia can result from increased purine catabolism due to tissue hypoxia in COPD patients [33] since tissue hypoxia can stimulate the biosynthesis of purine nucleotides [34,35]. Shaaban reported that modifications of purine with pyrazole moiety resulted in antioxidant activity and that this had potential in anticancer therapy [36]. Another hypothesis is that an elevated level of uric acid acts as an antioxidant in ROS-activated COPD patients [33]. The mechanism of hyperuricemia in COPD patients has yet to be clarified. However, our study strengthens the hypothesis that hyperuricemia is correlated to patients with obstructive lung disease.

In addition, a significant decrease in renal function was also observed in patients with FEV1/FVC < 70%. Crosstalk between COPD and renal function can attribute to a complex mechanism containing inflammation [37,38]. The inflammatory mediators affect both lung and renal function [39]. Klein et al. conducted a study on mice to explore myeloperoxidase activity of the lungs after renal injury or nephrectomy. The result suggested an increase in pro-inflammatory factors such as interleukin (IL)-6 correlated to impaired renal function [40]. Ahuja et al. [41] further studied how IL-6 affected lung function. After injection of the IL-6 factor to IL-6-deficient mice, a decrease in lung neutrophils and C-X-C motif chemokine ligand 1 (CXCL-1) was observed. Their animal study suggested that IL-6-mediated lung injury was associated with CXCL-1 production [41]. Moreover, acute kidney injury-mediated lung injury was caused by T lymphocyte activation, leading to apoptosis of lung epithelial cells and barrier dysfunction via the caspase-3 mechanism [42]. Our findings behaved as clinical evidence that corresponded to previous studies.

Lastly, a significant reduction in hemoglobulin levels was observed in patients with FEV1/FVC < 70%. Though anemia and COPD were reported to co-exist, the exact correlation and casualty are under debate [43,44,45]. Among all the causes of anemia, anemia of chronic disease is considered closely related to COPD, yet the mechanism has been explored by John et al. in 101 patients with COPD. They found patients with COPD and anemia also have elevated erythropoietin (EPO) levels, suggesting EPO resistance in COPD is related to inflammation [46]. The regulation of hepcidin production, an anemia factor, is another mechanism of COPD and anemia crosstalk. Many studies have shown that hepcidin production is induced by increased proinflammatory factors [47,48]. Our finding of decreased hemoglobulin levels in COPD patients can galvanize COPD and anemia crosstalk. A conclusive mechanism will be explored in our next clinical study.

The main strength of this study is the large-scale investigation of the association between betel nut chewing and its characteristics with lung function. However, there are also several limitations to the present study. First, this was a cross-sectional study, and we did not evaluate the duration of lung disease in the study patients. Consequently, causal relationships between chewing betel nut chewing and lung disease could not be evaluated. Nonetheless, our results may provide evidence of the importance of betel nut chewing on obstructive lung disease in Taiwan. Further longitudinal studies are warranted to clarify this issue. Second, we did not have data on the use of medications, and certain medications may have affected lung function. Third, we lack the results of the airway reversibility test to explain the possible mechanisms. In addition, further detailed assessment of betel nut chewing should have been more precise, including the daily amount with the equivalent in international units, the form of use, and the processing methods regarding betel nut. Finally, all of the participants in this study were of Chinese ethnicity, and thus our findings may not be generalizable to other ethnicities.

## 5. Conclusions

In conclusion, in this large population-based cohort study, we found that chewing betel nuts was associated with obstructive lung disease. Furthermore, a long duration of betel nut chewing, more frequent use, higher daily amount, and high cumulative dose were associated with obstructive lung disease. This suggests that preventing betel nut chewing should be considered to prevent obstructive lung disease in Taiwan.

## Figures and Tables

**Table 1:** Comparison of clinical characteristics among participants according to FEV1/FVC ≥ 70% or <70%.

Characteristics	FEV1/FVC ≥ 70% (*n* = 65,199)	FEV1/FVC < 70% (*n* = 15,678)	*p*-Value
Age (year)	49.7 ± 10.9	50.6 ± 11.0	<0.001
Male gender (%)	37.7	35.4	<0.001
Smoking history (%)	28.4	27.8	0.167
DM (%)	5.0	5.1	0.601
Hypertension (%)	12.0	12.5	0.090
Asthma (%)	4.2	3.6	0.001
Emphysema or bronchitis (%)	1.2	1.5	0.011
Betel nut chewing history (%)	6.3	6.7	0.076
Regular exercise habit (%)	40.4	43.8	<0.001
BMI (kg/m^2^)	24.3 ± 3.8	23.9 ± 3.6	<0.001
Laboratory parameters			
White blood cell (*10^3^/uL)	5.8 ± 1.6	5.9 ± 1.6	<0.001
Hemoglobin (g/dL)	13.8 ± 1.6	13.7 ± 1.6	<0.001
Fasting glucose (mg/dL)	95.9 ± 20.1	95.5 ± 20.3	0.061
Triglyceride (mg/dL)	117.1 ± 93.8	112.2 ± 83.6	<0.001
Total cholesterol (mg/dL)	195.8 ± 35.9	195.1 ± 35.0	0.034
HDL-C (mg/dL)	54.5 ± 13.5	54.4 ± 13.3	0.511
LDL-C (mg/dL)	121.1 ± 31.8	120.9 ± 31.4	0.542
eGFR (mL/min/1.73 m^2^)	35.3 ± 46.8	33.3 ± 46.5	<0.001
Uric acid (mg/dL)	5.4 ± 1.4	5.4 ± 1.4	0.584
Lung function			
FVC (L)	2.83 ± 0.80	2.75 ± 0.84	<0.001
FEV1 (L)	2.48 ± 0.71	1.40 ± 0.57	<0.001
FEV1/FVC (%)	87.8 ± 7.1	50.9 ± 13.7	<0.001

Abbreviations. FVC, forced vital capacity, FEV1, forced expiratory volume in 1 s; DM, diabetes mellitus; BMI, body mass index; HDL-C, high-density lipoprotein cholesterol; LDL-C, low-density lipoprotein cholesterol; eGFR, estimated glomerular filtration rate.

**Table 2:** Association of betel nut chewing history with FEV1/FVC < 70% in all study participants (*n* = 80,877) using multivariable logistic regression analysis.

Variables	Multivariable (FEV1/FVC < 70%)	
	Odds Ratio (95% CI)	*p*-Value
Betel nut chewing history	1.159 (1.072–1.254)	<0.001
Age (per 1 year)	1.007 (1.006–1.009)	<0.001
Male (vs. female)	0.645 (0.550–0.755)	<0.001
Smoking history	1.017 (0.970–1.067)	0.482
DM	0.982 (0.896–1.077)	0.700
Hypertension	1.042 (0.984–1.104)	0.163
Asthma	0.857 (0.781–0.941)	0.001
Emphysema or bronchitis	1.190 (1.025–1.383)	0.022
Regular exercise habit	1.107 (1.066–1.150)	<0.001
BMI (per 1 kg/m^2^)	0.961 (0.955–0.966)	<0.001
Laboratory parameters		
White blood cell (per 1 × 10^3^/uL)	1.080 (1.068–1.092)	<0.001
Hemoglobin (per 1 g/dL)	0.967 (0.953–0.981)	<0.001
Fasting glucose (per 10 mg/dL)	1.000 (0.999–1.001)	0.694
Triglyceride (per 10 mg/dL)	0.999 (0.998–0.999)	<0.001
Total cholesterol (per 10 mg/dL)	1.002 (1.000–1.005)	0.034
HDL-C (per 10 mg/dL)	0.991 (0.988–0.994)	<0.001
LDL-C (per 10 mg/dL)	0.998 (0.996–1.000)	0.052
eGFR (per 1 mL/min/1.73 m^2^)	1.003 (1.002–1.005)	<0.001
Uric acid (per 1 mg/dL)	1.053 (1.036–1.070)	<0.001

Values expressed as odds ratio and 95% confidence interval (CI). Abbreviations are the same as in Table 1.

**Table 3:** Comparison of betel nut chewing characteristics among participants with betel nut chewing history according to FEV1/FVC ≥ 70% or <70%.

Betel Nut Chewing Characteristics	FEV1/FVC ≥ 70% (*n* = 4103)	FEV1/FVC < 70% (*n* = 1032)	*p*-Value
Years of chewing betel nut (years)	14.0 ± 11.1	15.1± 10.9	0.002
How often do you chew betel nut?			0.012
1–3 days/month (score = 1)	7.4	4.5	
1,2 days/week (score = 2)	15.4	11.8	
3–5 days/week (score = 3)	16.2	13.8	
Everyday (score = 4)	61.0	69.9	
How many betel nuts a day?			0.005
<10 quids (score = 1)	41.1	31.2	
10–20 quids (score = 2)	34.2	37.4	
21–30 quids (score = 3)	11.1	13.8	
≥31 quids (score = 4)	13.6	17.7	
Cumulative dose (year × frequency × daily)	119.6 ± 132.7	148.2 ± 148.2	0.001

Abbreviations. FVC, forced vital capacity, FEV1, forced expiratory volume in 1 s.

**Table 4:** Association of betel nut chewing characteristics with FEV1/FVC < 70% in study participants with betel nut chewing history (*n* = 5135) using multivariable logistic regression analysis.

Variables	Multivariable (FEV1/FVC < 70%)	
	Odds Ratio (95% CI)	*p*-Value
Years of chewing betel nut (per 1 year) #	1.008 (1.002–1.014)	0.012
How often do you chew betel nut? #		
1–3 days/month (score = 1)	Reference	
1, 2 days/week (score = 2)	1.245 (0.668–2.323)	0.490
3–5 days/week (score = 3)	1.346 (0.729–2.484)	0.342
Everyday (score = 4)	1.793 (1.038–3.097)	0.036
How many betel nuts a day? #		
<10 quids (score = 1)	Reference	
10–20 quids (score = 2)	1.404 (1.058–1.862)	0.019
21–30 quids (score = 3)	1.662 (1.130–2.446)	0.010
≥31 quids (score = 4)	1.717 (1.199–2.459)	0.003
Cumulative dose (per 1 year × frequency × daily) #	1.001 (1.001–1.002)	0.002

Values expressed as odds ratio and 95% confidence interval (CI); # Adjusted for age, sex, smoking history, DM, hypertension, asthma, emphysema or bronchitis, regular exercise sport, BMI, white blood cell, hemoglobin, fasting glucose, triglyceride, total cholesterol, HDL-C, LDL-C, eGFR, and uric acid.

## Data Availability

The data underlying this study is from the Taiwan Biobank. Due to restrictions placed on the data by the Personal Information Protection Act of Taiwan, the minimal data set cannot be made publicly available. Data may be available upon request to interested researchers. Please send data requests to: Szu-Chia Chen, Division of Nephrology, Department of Internal Medicine, Kaohsiung Medical University Hospital, Kaohsiung Medical University.
